# Effectiveness of a tele-rehabilitation intervention to improve performance and reduce morbidity for people post hip fracture - study protocol for a randomized controlled trial

**DOI:** 10.1186/s12877-019-1141-z

**Published:** 2019-05-20

**Authors:** Yafit Gilboa, Talia Maeir, Sharon Karni, Michal E. Eisenberg, Meir Liebergall, Isabella Schwartz, Yakir Kaufman

**Affiliations:** 1 School of Occupational Therapy, Faculty of Medicine, Hadassah and the Hebrew University of Jerusalem, Mount Scopus, 91240 Jerusalem, Israel; 2 Herzog Medical Center, Givat Shaul, P.O.Box 3900, 91035 Jerusalem, Israel; 3 Hadassah University Hospital, P.O.Box 12000, 91120 Jerusalem, Israel

**Keywords:** Hip fracture, Elderly, Tele-rehabilitation, Cost-effectiveness, In-home rehabilitation, Transition, Occupational therapy, Community dwelling, Problem-solving

## Abstract

**Background:**

Most surviving hip-fracture patients experience reduced mobility and lose some of their functional ability, which increases the risk of complications and rehospitalization. Post-discharge transitional programs to reduce readmissions and disabilities have shown some success. Telerehabilitation refers to the use of technologies to provide rehabilitation services to people in their homes. Considering the need for long-term follow-up care for people with hip fracture, in-home telerehabilitation could increase independence, decrease hospital stays and reduce the burden for caregivers. The objective of this study is to investigate the effectiveness of an intervention program based on telerehabilitation on activities of daily living (ADL), quality of life (QOL), depression and burden on caregivers compared to face-to-face home visits and usual care of community-dwelling older adults after hip fracture.

**Methods/design:**

This will be a three-armed randomized control trial (RCT) including pre/post intervention and follow-up. The trial will include 90 older people with hip fractures who will be randomly assigned to a telerehabilitation group (*N* = 30), face-to-face visits (N = 30) and a control group. The aim of the intervention is to improve the transition from rehabilitation units to community dwelling. It will include 10 videoconferencing/ face-to-face sessions from an occupational therapist in the presence of the primary caregiver. Each session will be utilized to guide the participants to achieve their self-identified goals, focusing on problem-solving for daily life situations and on the ability to implement the discussed strategies for a variety of activities. Outcome measures include Functional Independence Measure (FIM) for evaluation of ADL, SF-12 for evaluation of Health-related QOL, The Geriatric Depression Scale (GDS) and The Zarit Caregiver Burden Scale. Data will be analyzed using Repeated measures MANOVA.

**Discussion:**

The current study will enable the cost-effectiveness examination of a suggested rehabilitation service based on available technology. The proposed intervention will increase accessibility of in-home rehabilitation services, improve function and health, and reduce economic burden.

**Trial registration:**

NCT03376750 (12/15/2017).

## Background

Hip fracture is one of the most serious osteoporotic fracture, and the second most common cause of hospitalization among the age group older than 65 years [1]. Hip fractures occur predominantly in the geriatric population [2, 3], due to higher rates of osteoporosis and falls [4]. The lifetime risk of hip fracture is 9% for women and 4% for men [5]. Hip fractures are a major public health problem in terms of patient morbidity, mortality and costs to health and social care [4, 6]. With an ageing population, and a growing proportion of people aged 80 years or older, the societal and economic burden of osteoporosis-related fractures is deemed to be increasing [7]. The healthcare costs for hip fractures are the highest (€20,000/patient) in comparison with other costs of injuries [8]. Osteoporotic hip fractures incur a high economic cost mainly due to the first hospitalization [8], but also due to subsequent outpatient visits, home care [6] and complications following initial hospital discharge [4, 9]. Therefore, further research is needed to identify the most effective and cost-effective rehabilitation pathways for these patients [10].

A third of older adults with hip fracture will be readmitted to hospital within 30 days of their return home [11], and up to 20% of patients die in the first year following hip fracture [12]. Hip fractures have profound effects on quality of life (QoL) and activities of daily living (ADL) [13, 14]. Most patients surviving hip fracture experience reduced mobility, lose their functional ability [15] and independence [16], and suffer from depression [17]. A systematic review found that after 1 year, 29% of hip-fracture patients did not reach their pre-fracture level of ADL, and 35% were not able to walk independently [2].

Hip fractures nearly always necessitate hospitalization and rehabilitation [18, 19]. Rehabilitation is a significant factor impacting the level of functional independence following hip fracture [20]. In the United States, more than 90% of Medicare beneficiaries utilize post-acute-care rehabilitation after an acute hip fracture hospitalization [21]. In Israel, 73.5% of the hip-fracture patients received inpatient rehabilitation, 14.3% received ambulatory rehabilitation treatment without an inpatient phase, and 12.2% received no rehabilitation [22].

The objective of rehabilitation is successful community discharge— facilitating a safe return to the community, thereby minimizing the likelihood of long-term institutionalization or reentry into the healthcare system [21]. However, a substantial decline in functional ability of hip-fracture patients occurs 1 year post discharge from rehabilitation. More than half of the hip-fractured elderly patients were unable to maintain their rehabilitation achievements 1 year post discharge [23]. Thus, to be efficacious in reducing or reversing disability after hip fracture, rehabilitation needs to be individualized, include many components, be progressive, and span a sufficiently long period [24].

Transitions between healthcare settings are a high-risk period for care quality and patient safety, mainly for older patients – such as those with hip fracture – who have complex needs and may undergo multiple care transitions [25]. Particularly for older adults, transitions are often plagued with discontinuity and a lack of coordination [26]. Patterns and problems associated with transitions after hip fracture in older adults include weight loss, delirium, depression, pressure ulcers, falls, and urinary incontinence [27], resulting in increased use of hospital, ambulatory and emergency services [26]. Gaps in communication and timely delivery of information are commonly cited barriers for inter-professional teams working along the continuum of care [25]. Strategies to improve transitional care to older hip-fracture patients should include improved patient and family involvement at the time of transition [27], communication and information sharing [25].

To summarize, hip fractures are one of the most common and potentially devastating injuries amongst the geriatric population [1]. The incidence, morbidity, and medical costs associated with hip fracture among the elderly are well-recognized [20]. The economic burden associated with hip fractures calls for the investigation of innovative and cost-effective forms of services [28]. Healthcare providers should be aware of the necessity for an individualized, holistic, long-term post-rehabilitation intervention program to prevent functional decline [9, 23].

Traditional in-home therapy provides the opportunity for rehabilitation intervention to occur within the context of everyday task performance [29]. Moreover, home rehabilitation programs have positive long-term effects in comparison with conventional care after hip fracture in elderly people [30–33]. Nonetheless, although in-home rehabilitation has been generally successful in preventing ADL decline [34] and facilitating recovery of ADL functioning and locomotion [35], problems caused by distance, the cost of travel and absence of healthcare personnel limit its utility [36]. To overcome these limitations, several studies have demonstrated that in-home therapy delivered to the elderly using interactive video-conferencing can successfully treat ADL task-performance deficits [37–39]. These studies suggest that relatively inexpensive video-conferencing technology can maintain the patient-therapist interactions, and provide a pragmatic alternative to face-to- face home visits by a therapist for improving function [36] and reducing healthcare spending [39].

This “telerehabilitation” strategy for chronic and prolonged disease management involves the patient in the process of medical care, provides continuous medical monitoring and early symptoms detection allows the ability to promptly respond to an acute exacerbation [40]. In addition, it can help to lower the psychological threshold for accessing medical care in people who are not aware of the seriousness of their symptoms, or who might otherwise be reluctant to access face-to-face care [41]. The evolving telecommunications industry has been proposed as a realistic solution to reduce healthcare cost, time, hospital visits [42] and caregiver burden [43], and provide long-term and easily accessible remote medical services [39, 44]. By using telerehabilitation services, client access to care can be improved and the reach of clinicians expanded, thus enhancing the continuity of care to persons with disabling conditions [45, 46]. A study that compared the effectiveness of video-conferencing with traditional outpatient clinic visits in the implementation of the management plan showed that video-conferencing is a valid alternative for orthopedic specialist consultations [47]. The results of another study suggest that conducting pre-admission occupational therapy “home visits” via the Internet with patients who are scheduled to undergo a total hip or knee replacement is both feasible and accurate [48].

Recent studies demonstrated the potential of home-based telerehabilitation in older adults and individuals with mobility impairment [49, 50]. As far as we know, however, only one paper so far has aimed to assess the impact of an older patient’s acceptance of hip-fracture telerehabilitation. They found that this intervention protocol targeting individualized impairments was feasible, safe and effective for use in home-based rehabilitation of older adults after hip fracture [51]. However, this research included only 10 patients and employed a quasi-experimental pre/post design without control group or follow-up. A study with longer duration and larger sample size, which also includes follow-up, needs to be done to measure hip-fracture recovery outcomes including functional parameters.

Some papers have addressed the issue of cost-effectiveness of telerehabilitation services to older adults. The first research assessed the feasibility of providing home-based telerehabilitation as an alternative to traditional ambulatory rehabilitation in patients post stroke, fracture or long hospital admission. There was a 50% reduction in staff’s home visits due to the telerehabilitation intervention. The therapists were able to provide twice the amount of service and direct patient-contact time, whilst reducing by 50 % time they spent travelling [52]. In addition, the effectiveness of a virtual reality-based telerehabilitation program for balance recovery after stroke was compared with conventional physical therapy program in the clinic. Results showed that the clinic-based intervention had a higher cost relative to the virtual reality intervention [53]. An Israeli case study described novel integrated motor imagery treatment designed for the rehabilitation of walking, which was delivered in the home through telerehabilitation for a person after stroke. Compared with on-site delivery, the telerehabilitation sessions resulted in lower therapist travel time and cost, as well as shorter therapy sessions [54]. Economic analyses were conducted as part of trials using telerehabilitation for total knee arthroplasty [55, 56]. The cost for a single session of in-home telerehabilitation compared to conventional home-visit rehabilitation was lower or about the same, depending on the distance between the patient’s home and healthcare center. A favorable cost differential was observed when the patient was more than 30 km from the provider [56]. Even if transportation costs were excluded, telerehabilitation was still a cost-effective alternative to usual care [55].

To summarize, the idea of telerehabilitation is becoming a more concrete means to deliver services to patients in their home environment, allowing patients to gain self-confidence in their ability to manage their health conditions [46]. Recent studies demonstrated the potential of home-based telerehabilitation in older adults [47, 49, 50] and its cost-effectiveness [52, 53]. However, limited evidence exists on the feasibility of home-based telerehabilitation in older adults after hip fracture**.**

## Methods

### Study aims

The primary aim of this study is to compare the effectiveness of in-home telerehabilitation and face-to-face home visit intervention programs, as opposed to the standard care currently offered to community-dwelling older adults after hip fracture. The secondary aims of this study are: (1) to evaluate the maintenance of the interventions’ accomplishments 3 months after completion; (2) to estimate the cost-effectiveness of the intervention programs; and (3) to understand the user experience of the patients and caregivers.

### Trial design, randomization and recruitment

This study is a three-armed, evaluator-blind, randomized control trial (RCT) and will be implemented according to CONSORT guidelines [57]. Participants will be assigned to research and control groups by using a blocked randomized controlled design, stratified based on age. The randomization sequence will be computer generated and will be known only to the responsible investigator.

**Fig. 1:**
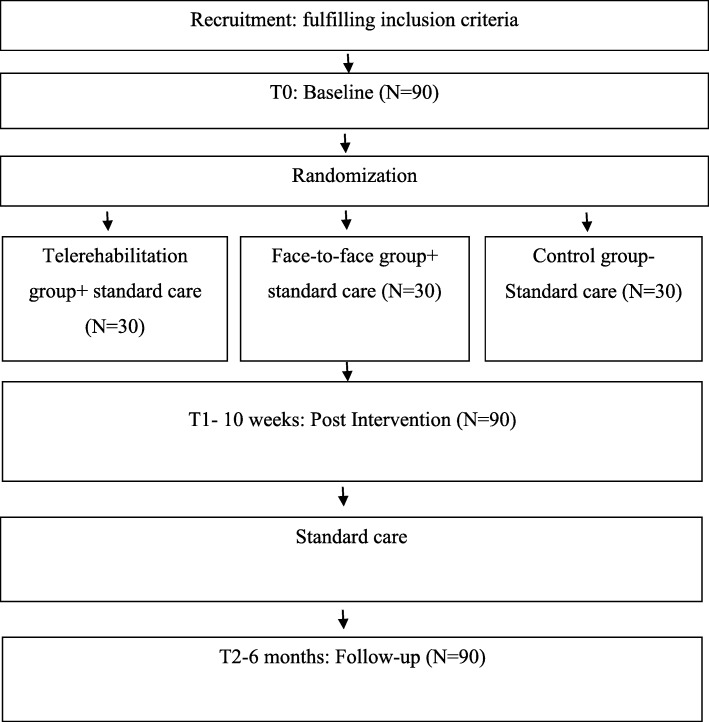
Schematic illustration of the study design

All participants will be recruited at the time of discharge from hospital. Health professionals employed at the hospital will screen and refer to a research assistant the interested clients who meet the inclusion criteria. The research assistant will obtain informed consent from caregivers and patients prior to randomization and trial inclusion. Participants (*n* = 90) will be randomized after baseline assessment in the three-arm study. The rehabilitation program will be identical in both intervention groups; only the delivery mode will differ: in-home telerehabilitation or face-to-face home visit. The patients allocated to all groups will continue to receive the standard usual care to improve adherence to intervention protocols. All outcomes will be measured at all three time points: Baseline-pretest (T0), immediately following intervention (post intervention 10 weeks) (T1), and a 6-month follow-up (T2) (See Fig. 1).

Recruitment and baseline testing will be carried out at Herzog and Hadassah Medical Centers, Jerusalem Israel. Intervention sessions will be conducted by video-conferencing or face-to-face visit while the participants are at their homes. Post-intervention and follow-up assessments will be carried out by e-mail or phone. The participation in the study will discontinue upon participants’ request.

### Study population

Participants in post-acute phases of recovery after hip fracture (ICD-10 code: S72.0) [58].

#### Inclusion criteria

Older adults (age ≥ 60 years) after hip fracture, who will be discharged from the rehabilitation or orthopedics units of Herzog and Hadassah Hospitals, Jerusalem, between January 2017 and December 2019. Discharge will be from rehabilitation to a non-institutionalized setting. Patients with broadband or Wi-Fi in their home and able to operate an iPad or computer independently. Functional Independence Measure [49] score at rehabilitation discharge: FIM > 90. Every participant will designate a caregiver (a close friend, family member, or support worker) aged over 18, who will agree to cooperate in the research and be present during the interventional phases to promote participants’ retention.

#### Exclusion criteria

Aphasia, cognitive impairment (Montreal Cognitive Assessment scores: MOCA< 21) [59], other degenerative neurological diagnoses, current major depressive or psychotic disorder, or other acute or chronic health condition that will influence their ability to participate in the study.

### Intervention

The aim of the intervention is to improve functional outcomes during the transition from rehabilitation units to community living and facilitate involvement in life roles in the community. The intervention program will be based on the Cognitive Orientation to daily Occupational Performance (CO-OP) [60]. CO-OP reflects a paradigm shift from component-focused rehabilitation traditionally used, to a broader, client centered, occupation based approach. CO-OP has the potential to support the long-term maintenance of therapeutic achievements following a rehabilitation and encourage the generalization and transfer of skills that will likely lead to continued improvement, rather than additional deterioration [61]. The strategy of CO–OP is to focus treatments directly on improving performance in everyday life activity, rather than treating the underlying impairments and hoping for secondary improvement in meaningful activities [62]. The consensus of the literature examining the CO-OP approach suggests that its use in many novel populations and settings is promising and is expanding [63].

CO-OP is a top-down, task-oriented, client-centered approach that uses an iterative process of dynamic performance analysis and guided discovery to enable individuals to identify strategies that will improve performance [63]. Studies that implement these principal elements in their intervention program described supportive results among elderly after hip fracture [30, 33, 64, 65]. The findings indicate that participating in treatment which focuses on individually chosen and personally meaningful activities improves quality of life [33], balance confidence, independence and physical activity [64] with long-term effect [30] and decreased length of hospital stays [65]. Additionally, previous research demonstrated the effectiveness of CO-OP on function-related goals in post-stroke elderly patients [61, 62, 66–68], which suggests that CO–OP may have a broader positive effect on recovery than conventional occupational therapy care [61, 62]. The older adult populations were comprised of those with no diagnosis of dementia, depression, or cognitive impairment upon testing [62, 63].

In line with previous protocol that included post-stroke elderly patients with a parallel cognitive profile [62], participants will be receiving 10 h of training (1-h sessions/week) from a trained occupational therapist. Through dialogues, the therapist directs the individual to discuss the task’s purpose (goal), identify strategies to achieve it (plan), execute these strategies (do), and evaluate their progress and outcome (check). The intervention will be performed by real-time video-conferencing using an iPad® and Skype™, a software program that allows video calls over the Internet. The CO-OP approach administered in a telerehabilitation format was found to be feasible for adults with traumatic brain injury [69].

#### Treatment fidelity

The occupational therapists will record digital audio or take notes each session. The data of the process and content of the intervention will be used to ensure the treatment fidelity of the intervention. Recordings of the first five sessions of the first 5 participants will be monitored. The occupational therapists providing the intervention and an additional research assistant will review the session recordings to ensure that the CO-OP is delivered as intended, following the essential aspects of intervention.

### Control group

The purpose of the control group is to control for changes that may occur with the passage of time in the estimate of the efficacy of the intervention [70]. This group will receive the usual care and rehabilitation by the common routine for patients with hip fracture, and will be discharged with no further therapy as part of the study. In addition, the clinicians will receive no direction from the research study staff.

### Measurements

#### Sample characterization

##### Demographic questionnaire

This will be used to describe self-reported medical and personal details like age, length of hospitalization, weight bearing status and medical services received post discharge.

### Outcome measures

#### Functional Independence Measure (FIM) [71]

The aim of the FIM is to monitor the recovery of functional ability by people undergoing rehabilitation. The FIM is comprised of 18 parameters, each rated on a scale of 1–7 (range = 18–126) according to the degree of assistance required to perform a specific activity.

#### 12-item MOS Short-Form Health Status Survey [72], Hebrew version [73]

This generic HR-QoL instrument focuses on functional status. The questionnaire includes 12 items taken directly from the SF-36 [74] which are used to calculate the Physical and Mental Component Summary. The first question measured by the SF-12 is an acceptable self-rated measure for general health [75].

#### The Geriatric Depression Scale (GDS)-Short [76], Hebrew version [77]

The scale consists of 15 items; each item has two possible answers (yes or no). The highest possible score is 15, which indicates the most severe depressive state.

*The Canadian Occupational Performance Measure (COPM)* [65] will be used for measuring performance and satisfaction with personally identified participation goals. Participants are asked to identify goals and then rate their performance and satisfaction with current status on a scale from 1 to 10, where 10 indicates optimal performance or satisfaction.

#### The Zarit Caregiver Burden Interview (ZBI) [78]

The ZBI includes 22 statements recorded in a 0–4 Likert scale (total score range 0 to 88, where higher scores mean higher burden), which rates the subjective component of burden.

#### Qualitative interview

Semi-structured interviews will be conducted post intervention with the patients in the research groups (*n* = 60) and their caregivers.

(n = 60) to identify barriers and facilitators regarding the telerehabilitation and face-to-face programs.

### Data analysis

Quantitative data will be analyzed using SPSS 21.0. Descriptive statistics will be obtained on the demographic, social and clinical pretest characteristics of subjects in the three groups. Differences in these variables between the intervention and control groups will be examined using either chi-square tests or analysis of variance. Feasibility in the research groups will be calculated according to the of participants who complete the trial.

Repeated-measures MANOVA will be used to test the effects of the group assignments (intervention vs. control), the effects of time (baseline, post-intervention, and 6-month follow-up), and the group-by-time interaction for measuring all the outcomes. Repeated-measures MANOVA is recommended as being straightforward, powerful, and appropriate to treatment outcome investigations [79]. In addition to providing a multivariate analysis of the effect of treatment on the combined changes in all measures and healthcare costs, this approach also provides a univariate analysis of the effect of treatment on each measure, thus allowing an investigation of which measures were most affected by treatment [79]. *p*-Values less than 0.05 are considered to be statistically significant in all comparisons.

All qualitative interviews (patients and caregivers) will be video/audio-recorded and transcribed. Conventional content analysis with constant comparative methods will be used for coding and analysis. All data will be collected anonymously, and access will be restricted to the investigators.

### Sample size calculation

The sample size was based on RCT that compared the effectiveness of face-to-face home rehabilitation to conventional care after hip fracture of geriatric patients [64]. The home rehabilitation group showed a higher degree of recovery in self-care using the FIM, (calculated effect size: d = 0.8). The minimum sample size at the significance level of 5% (one-sided test) and power of 80% is 25 in each group. Estimated drop-out rate is 20%; therefore, a maximum of 30 participants will be recruited for each group.

## Discussion

This study has the potential to guide clinical practice toward innovative modes of rehabilitation care and enhance clinical management of hip fracture in older people. If efficacious, our results will provide a novel online delivery method that rehabilitation professionals can incorporate into their treatment program, which in turn will optimize outcomes. The proposed innovative telerehabilitation directly addresses one of the major issues of geriatric rehabilitative care – continuity. It is designed to support the weakest links in the chain of care transition [26]. It will assist in the transition from hospital to community dwelling, as well as maintaining the rehabilitation achievements. Since the hours of supervised therapy are limited, telerehabilitation will enable patients to expand significantly the hours that they practice therapeutic exercises [54], while promoting more effective methods of long-term rehabilitation and maintenance of a healthy lifestyle [80].

There are barriers to overcome relating to patients’ personal restrictions, staff logistical issues of the systems to ensure the implementation of telerehabilitation more widely in older adults [81]. However, community-dwelling older people who have received a home telerehabilitation program found telerehabilitation convenient and motivating, coped well with the technology, and developed positive therapeutic relationships [82]. In addition, the current research will allow examination of the cost-effectiveness of telerehabilitation in a large cohort of patients after hip fracture. The availability of this service will enable reduction of the length of rehabilitation hospitalizations, and will be used as an alternative to conventional ambulatory rehabilitation, particularly for those patients in rural and peripheral areas. Furthermore, this type of service is compatible with the evolving models of self-management, in which clients are encouraged to take responsibility for managing their diseases or impairments [83].

To summarize, three major innovations are presented in our suggested protocol: First, this protocol outlines the justification for the first RCT study in telerehabilitation service for patients after hip fracture. Second, our proposed service is based on available, easy-to-use, inexpensive, simple technology [46], using off-the-shelf applications. Last, the focus is on individualized day-to-day function rather than exercising, as an individualized approach may be most effective due to the patient's unique characteristics [84].

## Abbreviations

ADL: Activities of daily living
CO – OP: Cognitive Orientation to daily Occupational Performance
COPM: The Canadian Occupational Performance Measure
FIM: Functional Independence Measure
GDS: Geriatric Depression Scale
MOCA: Montreal Cognitive Assessment
QOL: Quality of life
RCT: Randomized controlled trial
SF– 12: 12-item MOS Short-Form Health Status Survey
ZBI: The Zarit Caregiver Burden Interview.
